# A pH-Triggered antibacterial and lubricating dual-function hydrogel coating for infection-resistant urinary catheters

**DOI:** 10.3389/fbioe.2026.1751442

**Published:** 2026-01-21

**Authors:** Ming Cheng, Weijie Lin, Jianbo Yu, Peiliang Gao, Fange Shi, Chunyu Wang, Yong Ma, Zhongdi Liu, Guiying Dong

**Affiliations:** 1 Emergency Department, Peking University People’s Hospital, Beijing, China; 2 Trauma Treatment Center, Peking University People’s Hospital, Beijing, China; 3 Key Laboratory of Trauma Treatment and Neural Regeneration (Peking University) Ministry of Education, Beijing, China; 4 National Center for Trauma Medicine, Beijing, China

**Keywords:** antimicrobial peptides, catheter coating, hydrogel, magnesium oxide, pH-responsive antibacterial, sustained lubrication

## Abstract

**Introduction:**

Urinary catheterization frequently causes urinary tract infections and patient discomfort. While hydrogel coatings combining antibacterial and hydrophilic properties offer a potential solution, challenges such as uncontrolled antimicrobial release and poor coating adhesion limit their clinical utility. This study aimed to develop a novel dual-function hydrogel coating with controlled antibacterial activity and sustained lubrication for infection-resistant urinary catheters.

**Methods:**

A bilayer PL@SAMT/Mg coating consisting of an inner polydopamine layer loaded with the antimicrobial peptide LL-37 and an outer pH-responsive MgO@AAm/SA/TA hydrogel was fabricated via surface modification and UV crosslinking. The coating was applied to catheters via surface modification followed by UV-induced crosslinking. It was characterized using SEM, EDS, FTIR, rheometry, and friction tests. Its antibacterial efficacy was evaluated against Staphylococcus aureus and Escherichia coli at different pH levels. Cytocompatibility was assessed using CCK-8, live/dead staining, and ELISA assays with L929, SV-HUC-1, and RAW264.7 cells. In vivo biocompatibility and antibacterial performance were investigated using a rat subcutaneous implantation model.

**Results:**

The PL@SAMT/Mg coating exhibited a uniform, adherent bilayer structure with stable mechanical properties. It delivered excellent hydration lubrication and demonstrated pH-responsive swelling behavior. The release of LL-37 was sustained, while MgO release was significantly accelerated under alkaline conditions mimicking infection. The coating showed strong, pH-enhanced antibacterial activity against both S. aureus and E. coli. In vitro assays confirmed excellent cytocompatibility, anti-inflammatory effects, and anti-adhesion properties. In vivo, the coating minimized inflammation and significantly reduced bacterial colonization compared to uncoated catheters.

**Discussion:**

The PL@SAMT/Mg coating successfully integrates intelligent antibacterial function with sustained lubrication. The PDA layer enables long-term preventive release of AMPs, while the pH-responsive hydrogel layer provides on-demand MgO release during infection. This synergistic, controlled-release strategy mitigates biotoxicity and resistance risks. The coating addresses key limitations of existing technologies through robust adhesion, effective antibacterial action, and biocompatibility, offering a promising approach to improve catheter performance and patient comfort.

## 1 Introduction

The urinary catheter (UC) is one of the most widely used indwelling medical devices in clinical practice. It is extensively employed for managing urinary retention, caring for patients requiring prolonged bed rest due to conditions such as pelvic fractures, monitoring urine output in critically ill patients, and improving comfort in palliative care (Chantelle et al., 2019; Costa et al., 2020; Liu et al., 2021). However, the inherent lack of lubricity in catheter materials causes friction at the catheter–urethra interface during insertion, which can injure urethral tissue and cause patient discomfort (Zheng et al., 2024). Moreover, nearly all patients undergoing long-term indwelling catheterization develop infections (Priyadarshini et al., 2017). The mechanisms of catheter-associated urinary tract infection (CAUTI) are multifaceted. The strong hydrophobic surface of indwelling catheters facilitates bacterial adhesion and biofilm formation (Yu H. et al., 2019). Simultaneously, the catheter’s presence disrupts the urethra’s natural flushing defense, allowing bacteria to migrate retrogradely along the catheter surface (Stickler, 2014). Structural deficiencies in the catheter balloon design also lead to residual urine retention in the bladder, creating a favorable environment for bacterial proliferation (Drake et al., 2024). Although previous studies have attempted to design novel catheters, structural modifications alone have proven insufficient to prevent biofilm formation on catheter surfaces during long-term use (Ionescu et al., 2021; Levering et al., 2016; Sun et al., 2011). Consequently, recent research has increasingly focused on developing coatings and antimicrobial materials to address catheter-related pain and infection. Among these strategies, bifunctional coatings that combine hydrophilicity and antimicrobial properties on catheter surfaces have emerged as a promising approach (Siddiq and Darouiche, 2012; Yu et al., 2021). However, challenges such as the uncontrolled release of antibacterial agents and the poor adhesion stability of coatings remain unresolved (You et al., 2025; Hu et al., 2023). Therefore, it is crucial to design novel coatings with controllable antibacterial activity and sustained lubricating performance.

Release-based antibacterial materials represent a common strategy for imparting antibacterial functionality to urinary catheters (Ricardo et al., 2020). Currently, silver-based and antibiotic-based release-type bactericidal materials have been commercialized for the fabrication of antibacterial urinary catheters (Ricardo et al., 2020; Yang et al., 2024). However, the potential toxicity associated with the *in vivo* accumulation of silver ions and the emergence of drug resistance resulting from antibiotic overuse have limited their long-term safe application (Wang et al., 2021; Bai et al., 2023). In recent years, novel antibacterial materials such as magnesium oxide (MgO) and antimicrobial peptides (AMPs) have garnered widespread attention due to their low biotoxicity and excellent antibacterial properties (Coelho et al., 2020; Magana et al., 2020). Magnesium oxide nanoparticles (MgO NPs) exert unique bactericidal effects by generating reactive oxygen species (ROS), including hydroxyl radicals, superoxide anions (•O_2_^−^), and hydrogen peroxide, which damage bacterial deoxyribonucleic acid (DNA) and cellular proteins (Karthikeyan et al., 2021). Owing to this distinctive antibacterial mechanism, MgO, as an inorganic antibacterial material, is often combined with bioactive molecules to enhance its antibacterial efficacy. As multifunctional bioactive molecules, AMPs are regarded as a suitable alternative for combating multidrug-resistant bacteria due to their unique antibacterial mechanisms and potent sterilization capabilities (Magana et al., 2020). Membrane permeabilization represents the primary mechanism of AMP antibacterial action. The positively charged hydrophilic groups of AMPs can electrostatically adsorb onto the negatively charged bacterial cell wall surface, while hydrophobic residues insert into the bacterial membrane, leading to leakage of cellular contents and ultimately resulting in cell death (Xuan et al., 2023). Nevertheless, the clinical application of AMPs is primarily hindered by the potential biotoxicity resulting from their burst release (Liu et al., 2024). In recent years, polydopamine (PDA) coating, as a simple and mild surface modification method, has been employed in various antibacterial contexts to construct loading and delivery systems for AMPs. Previous studies have demonstrated that loading the CWR11 antimicrobial peptide onto PDA nanoparticles (PDA NPs) enables photothermally responsive and targeted controllable antibacterial functions (Andoy et al., 2020). Similarly, using a PDA coating to load the antimicrobial polypeptide poly(phenylalanine_10_-stat-lysine_12_) has been shown to effectively enhance the antibacterial performance of orthopedic implants without inducing antimicrobial resistance (Liu et al., 2023). However, to date, no relevant reports have been published on the utilization of PDA surface modification technology for the loading and long-term sustained release of AMPs to achieve controllable antibacterial performance in urinary catheters.

Owing to their high hydrophilicity and softness, hydrogels are frequently employed as urinary catheter coatings to improve lubrication (Xu et al., 2022; Yu Y. et al., 2019). However, common catheter materials such as silicone and polyvinyl chloride possess few active hydrophilic groups on their surfaces, which prevents strong bonding with hydrogel coatings (Faucheux et al., 2004). Appropriate surface modification of catheters is therefore essential for achieving robust hydrogel adhesion. Sodium alginate (SA), a natural polysaccharide, exhibits excellent biocompatibility and hydrophilicity (Vatanpour et al., 2022). It contains abundant carboxyl and hydroxyl groups that enable facile ionic crosslinking in aqueous solutions, making SA an ideal base material for lubricating coatings (Vatanpour et al., 2022). Acrylamide (AAm), a synthetic monomer, can form a polyacrylamide (PAAm) network through free radical polymerization. This network interacts with SA through hydrogen bonding or covalent crosslinking, forming a dual-crosslinked network hydrogel that further enhances the stability and toughness of the hydrogel (Zhang Y. et al., 2023). Methacrylic acid (MAA), a functional monomer rich in carbon-carbon double bonds, participates in free radical polymerization and further increases the crosslinking density between SA and AAm (Pu et al., 2024). Tannic acid (TA), a plant-derived polyphenolic compound, contains multiple functional groups capable of forming hydrogen bonds and hydrophobic interactions with various polymers (Zhang X. et al., 2023). This property enables TA to copolymerize with SA and PAAm, forming a three-dimensional hydrogel network that improves the coating’s adhesion to the catheter surface (Jafari et al., 2022). Ultraviolet (UV) crosslinking provides an efficient and mild curing method (Huang et al., 2011; Reddy et al., 2015), which can simultaneously induce ionic crosslinking of SA and free radical copolymerization of AAm on the catheter surface. This process yields a stably bonded composite coating (Xu et al., 2022), which effectively resists detachment during catheter use. Consequently, combining surface chemical modification with photo-initiated polymerization is crucial for enhancing the binding stability between coating and catheter. Furthermore, to mitigate the toxicity associated with uncontrolled release of metal-based antimicrobial agents, this study also utilizes the swelling characteristics of hydrogels to achieve pH-responsive antibacterial functionality. The MgO-loaded AAm/SA/TA hydrogel contains abundant carboxyl groups. The swelling behavior of this hydrogel varies with pH because carboxyl group ionization increases under alkaline conditions, whereas swelling is suppressed in acidic environments (Lei et al., 2025). During bacterial infection in the urinary tract, certain bacteria such as *Proteus* species can produce urease, which decomposes urea into ammonia, leading to urine alkalization (Miao et al., 2023). This alkalization promotes hydrogel swelling and accelerates MgO release, thereby enabling pH-responsive antibacterial action.

Recent research on functionalized urinary catheter coatings has increasingly emphasized surface modifications that integrate intelligent antibacterial properties with durable lubrication. pH-responsive coatings, which release antibacterial agents in response to changes in the infectious microenvironment, have garnered particular interest. For instance, Miao et al. (2023) developed a polyzwitterion–tannic acid hydrogel coating that rapidly releases antibacterial drugs under alkaline conditions, demonstrating effective pH-responsive antibacterial activity. In the design of antibacterial materials, novel agents such as AMPs and MgO have been innovatively applied. Yao et al. (2022) covalently grafted AMPs onto polyurethane ureteral stents using Cu^2+^-assisted polydopamine, achieving durable antibacterial and anti-encrustation functions. Meanwhile, MgO nanoparticles have been used to form effective antibacterial coatings on thermoplastic polyurethane catheter surfaces (Padrão et al., 2025). In terms of lubrication, Zheng et al. (2024) created a xylitol-based mucus-mimetic hydrogel coating with excellent lubricating and anti-biofouling properties. Furthermore, composite coatings that integrate quaternary ammonium salts with zwitterionic polymers have shown synergistic anti-biofilm effects (Bai et al., 2023). Despite these advances, current research still faces several challenges, including insufficient synergy between antibacterial and lubricating functions, limited long-term stability of coatings, and a lack of capability for intelligently switching antibacterial modes under varying environmental conditions. To address these limitations, we developed a PDA/LL-37-MgO@AAm/SA/TA (PL@SAMT/Mg) composite coating based on a bilayer architecture. Bilayer systems are increasingly prominent in medical device surface modification, as they can integrate distinct functional layers to achieve multifunctional performance. For instance, Shymborska et al. (2025) recently reported a responsive “smart” polymer sandwich structure that meets specific cellular requirements while retaining stimuli-responsiveness. Drawing on this concept, the PL@SAMT/Mg coating adopts a bilayer architecture to simultaneously fulfill the dual functions of intelligent responsive antibacterial activity and sustained lubrication. Specifically, the PL@SAMT/Mg coating consists of an inner polydopamine layer for loading AMPs to provide preventive, sustained release during non-infected periods, and an outer pH-responsive MgO@AAm/SA/TA hydrogel layer that accelerates MgO release under infectious conditions. The bilayer architecture simultaneously ensures stable lubrication and firm coating adhesion under wet conditions, offering an integrated solution that combines intelligent antibacterial responsiveness with sustained lubrication.

In this study, an MgO@AAm/SA/TA hydrogel coating was fabricated using ultraviolet cross-linking technology and tightly bonded to the catheter, while polydopamine (PDA) surface modification was employed to achieve loading and sustained release of the antimicrobial peptide LL-37. Material characterization and antibacterial experiments demonstrated that the PL@SAMT/Mg-coated catheter exhibited sustained lubrication and controllable antibacterial properties. Both *in vitro* and *in vivo* experiments also indicated favorable cytocompatibility and biocompatibility. Therefore, PL@SAMT/Mg represents a promising catheter coating material with stable low friction and controlled antimicrobial functionality, showing broad potential for clinical application.

## 2 Materials and methods

### 2.1 Materials

Sodium Alginate (SA), Acrylamide (AAm), Methacrylic Acid (MAA), Tannic Acid (TA), N,N'-Methylenebisacrylamide (MBA), N,N,N',N'-Tetramethylethylenediamine (TEMED), Ammonium Persulfate (APS), and Calcium Sulfate (CaSO_4_) were purchased from Shanghai Macklin Biochemical Technology Co., Ltd. Dopamine powder was obtained from Beijing McLean Reagent Co., Ltd. Antimicrobial peptide LL-37: Purity ≥ 98%, source batch = HY-P4744-202306 (Shanghai Chupeptide Biotechnology). *Staphylococcus aureus* (CMCC 26003) and *Escherichia coli* (ATCC 25922) were provided by the Shanghai Conservation Biotechnology Center. L929 cells, RAW264.7 cells, and SV-HUC-1 cells were acquired from the Chinese Academy of Sciences Beijing Cell Bank. SD rats aged 6–8 weeks and weighing approximately 200 g, were provided by Beijing Weitong Lihua Experimental Animal Technology Co., Ltd. Medical catheter substrate (thermoplastic polyurethane, TPU): Model = 24G (Nanchang Kanghua Medical Supplies), surface roughness Ra = 0.2 ± 0.05 μm (measured by a profilometer), surface functional groups = negligible hydrophilic groups (consistent with FTIR characterization). MgO nanoparticles: Particle size distribution = 50–100 nm, purity = 99.9% (provided by XFNANO Materials Technology Co., Ltd.), Crystal Violet and 4% Paraformaldehyde Fix Solution were purchased from Beijing Soleibao Biological Reagent Co., Ltd. Isoflurane (RWD Life Science Co., Ltd., Shenzhen, China). All other reagents and consumables were procured from Shanghai Macklin Biochemical Technology Co., Ltd.

### 2.2 Instrumentation equipment

SEM(HITACHI SU8010, Tokyo, Japan), EDS (HITACHI SU8010, Tokyo, Japan), FTIR (Thermo Scientific, Nicolet6700, USA), Rheometer (Waters DHR-2,USA), Friction and wear testing machine (Bruker(CETR)UMT-2), Inductively Coupled Plasma Mass Spectrometry(ICP-MS) (Shimadzu, ICPE-9800, Japan), Micro BCA Protein Assay Kit (Thermo Fisher Scientific), CCK8 kit (Dojindo, Tokyo, Japan), enzymoleter (SpectraMaxM2, Molecular Devices, Sunnyvale, CA, USA), Live/Dead cell staining reagents(Invitrogen, L3224, USA), Confocal laser microscope (OLYMPUS FV1000, Tokyo, Japan). TNF-αELISA and IL-6 ELISA were purchased from Shanghai Enzyme-linked Biotechnology Co., Ltd.

### 2.3 Synthesis of materials

#### 2.3.1 Synthesis of (AAm/SA/TA) hydrogels

At room temperature, 2.03 g of acrylamide (AAm), 0.34 g of sodium alginate (SA), 0.05 g of tannic acid (TA), and 0.15 g of methacrylic acid (MAA) were sequentially dissolved in 10.0 mL of deionized water under continuous stirring for 6 h. Next, 0.0012 g of N,N'-methylenebisacrylamide (MBA), 5 mg of N,N,N',N'-tetramethylethylenediamine (TEMED), 20.3 mg of ammonium persulfate (APS), and 44.7 mg of calcium sulfate were introduced into the solution and thoroughly mixed to yield a pre-gel solution. This pre-gel solution was crosslinked into a hydrogel upon ultraviolet (UV) light irradiation. The resulting hydrogel was then rinsed extensively with deionized water to eliminate unreacted species.

#### 2.3.2 Construction of the MgO@AAm/SA/TA hydrogel coating

Twenty milligrams of magnesium oxide nanoparticles were added to the pre-gel solution under stirring. The catheter was alternately ultrasonicated in isopropanol and deionized water for 15 min, dried with nitrogen, and then fully immersed in the mixture. Following removal, ultraviolet cross-linking was performed to solidify the coating on the catheter surface. The catheter was rinsed three times with deionized water to yield the MgO@AAm/SA/TA hydrogel-coated catheter.

#### 2.3.3 Construction of the PDA/AMPs coating

The medical catheter was cut into 1 cm segments, which were alternately cleaned ultrasonically in ethanol and deionized water for 15 min each before being dried under a nitrogen flow. These segments were then fully immersed in a 2 mg/mL dopamine hydrochloride solution and incubated at 37 °C with shaking at 120 rpm for 24 h to facilitate the spontaneous polymerization of polydopamine (PDA) on the catheter surface. After incubation, the samples were gently rinsed three times with deionized water.

The PDA-modified catheter was immersed in an antimicrobial peptide (LL-37) solution (100 μg/mL, dissolved in PBS, pH 7.4) and incubated at 37 °C with shaking at 120 rpm for 4 h, enabling peptide immobilization on the surface through adhesion to the PDA layer. The catheter was then rinsed three times with phosphate-buffered saline (PBS) to eliminate unbound antimicrobial peptides. This process yielded the Catheter@PDA–Antimicrobial Peptide substrate, which was stored at 4 °C for subsequent use.

The modified urinary catheter was then immersed in an MgO@AAm/SA/TA pre-gel solution for 5 min to achieve a uniform coating, after which it underwent ultraviolet (UV) light-induced crosslinking. UV crosslinking was performed using a UV crosslinker (Model: UVP CL-1000) at a wavelength of 365 nm, an irradiation time of 1 min, and a light intensity of 10 mW/cm^2^. This treatment enabled both the ionic crosslinking of SA and the free-radical copolymerization of AAm, producing a stable composite coating. The final product was rinsed three times with sterile deionized water for 5 min each and dried under nitrogen flow, yielding a pH-responsive composite coating material designated as “PDA/antimicrobial peptide–MgO@AAm/SA/TA” (abbreviated as PL@SAMT/Mg).

### 2.4 Chemical mechanisms underlying the fabrication of the PL@SAMT/Mg bilayer coating

The preparation of the PL@SAMT/Mg bilayer coating relies on synergistic chemical reactions and intermolecular interactions, which determine the coating’s structure, stability, and functional properties.

#### 2.4.1 Chemical formation mechanism of the PDA/AMPs layer

The inner layer forms *via* dopamine self-polymerization followed by immobilization of the antimicrobial peptide LL-37. In an oxygen-rich environment, the catechol groups in dopamine oxidize to quinones, which then undergo nucleophilic addition, Michael addition, and Schiff base reactions to gradually assemble a three-dimensional polydopamine network. This catalyst-free polymerization proceeds under mild conditions, and the resulting PDA adheres firmly to the catheter surface through both covalent and non-covalent interactions. The PDA surface, rich in catechol, amino, and imino groups, forms hydrogen bonds with the amide bonds and carboxyl groups of LL-37. Furthermore, π-π stacking occurs between aromatic rings in PDA and hydrophobic aromatic residues in LL-37, ensuring stable peptide loading and enabling its long-term, sustained release.

#### 2.4.2 Chemical crosslinking mechanism of the MgO@AAm/SA/TA hydrogel layer

The outer hydrogel layer forms a stable hydrogel network with uniformly dispersed MgO nanoparticles through a dual crosslinking system involving ionic crosslinking and free radical copolymerization.

The pre-gel solution contains SA, AAm, MAA, TA, and MgO nanoparticles. SA provides carboxyl groups for ionic crosslinking, AAm and MAA supply C=C bonds for free radical polymerization, and TA offers polyphenolic hydroxyl groups for hydrogen bonding. MgO nanoparticles are initially dispersed *via* hydrogen bonds between their surface hydroxyl groups and the polyphenolic hydroxyls of TA. The CaSO_4_ added to the pre-gel solution dissociates to release Ca^2+^ ions. The carboxyl groups on the SA molecular chains coordinate with Ca^2+^, forming an ionically crosslinked network that constructs the physical skeleton of the hydrogel.

Using APS as the initiator and TEMED as the accelerator under UV irradiation, APS decomposes to generate free radicals. These radicals subsequently initiate the free radical copolymerization of the C=C bonds in AAm and MAA molecules. This reaction creates a covalent P(AAm-co-MAA) network that interpenetrates the ionic SA network, yielding a dual-crosslinked hydrogel with enhanced mechanical strength and structural stability.

TA polyphenolic hydroxyl groups form hydrogen bonds with SA hydroxyls and P(AAm-co-MAA) amide bonds, further densifying the network. Hydrophobic interactions between TA and the polymer chains improve the coating’s adhesion to the catheter. Hydrogen bonds between TA polyphenolic hydroxyls and MgO surface hydroxyls also maintain nanoparticle dispersion within the hydrogel, preventing agglomeration and preserving antibacterial activity.

#### 2.4.3 Interfacial bonding mechanism between the bilayer coatings

The PDA/AMPs layer and the MgO@AAm/SA/TA hydrogel layer achieve robust bonding through hydrogen bonds and π-π stacking interactions. The catechol and amino groups on the PDA surface form strong hydrogen bonds with carboxyl groups of SA, amide bonds of P(AAm-co-MAA), and polyphenolic hydroxyls of TA in the hydrogel. Concurrently, π-π stacking occurs between aromatic rings in PDA and benzene rings in TA, which enhances the interfacial bonding strength.

### 2.5 Material characterization

#### 2.5.1 SEM and energy dispersive spectroscopy

A section of the coated catheter, approximately 2 × 2 mm in size, was excised. After vacuum drying, the sample was mounted on a specimen stub, sputter-coated with gold, and then examined using a field emission scanning electron microscope (SEM) at an accelerating voltage of 15 kV to observe the coating’s surface morphology. Micrographs were recorded with a 2 μm scale bar.

Energy dispersive spectroscopy (EDS) was used to analyze the distribution of carbon (C), oxygen (O), and nitrogen (N) from sodium alginate (SA), along with magnesium (Mg) as the characteristic element of MgO, within the coating. The cross-sectional profile of the coating and its corresponding EDS elemental maps were acquired at a 50 μm scale bar.

#### 2.5.2 Fourier transform infrared (FTIR) characterization

Dry AAm/SA/TA hydrogel samples were compressed into tablets for analysis. Attenuated Total Reflectance Fourier Transform Infrared (ATR-FTIR) spectroscopy characterized these samples directly in ATR mode. Each spectrum was collected from 32 accumulated scans at a resolution of 4.0 cm^-1^, covering the wavenumber range of 4,000–4,600 cm^−1^. The analysis focused on characteristic functional groups, including the carboxyl group of SA, the phenolic hydroxyl group of TA, and the amide bond of AAm, as well as the interaction peaks between MgO and the gel network.

#### 2.5.3 Swelling ratio

The AAm/SA/TA hydrogels and MgO@AAm/SA/TA hydrogels of equal mass were weighed and recorded, then immersed in PBS. At predetermined time points (0 min, 1 min, 5 min, 10 min, 30 min, 1 h, 2 h, 3 h, 6 h, 12 h, 24 h), the hydrogels were removed, and surface moisture was absorbed using filter paper before re-weighing. The swelling ratio of the samples was subsequently calculated.

$$\mathrm{Swelling ratio}\left(\%\right)=\frac{W_{s}-W_{d}}{W_{d}}\times 100\%$$

Here, W_d_ represents the initial weight of the hydrogel, and W_s_ represents the weight of the hydrogel after swelling.

#### 2.5.4 Rheological properties, friction coefficient, and Young’s modulus

A 20 μL hydrogel sample was analyzed using a modular compact rheometer (Waters DHR-2,USA) to determine the storage modulus (G′) and loss modulus (G″) as indicators of gel elasticity. Measurements were conducted at 37 °C under a shear stress of 1 Pa, with the frequency swept from 0.1 to 100 rad/s. The hydrogel underwent 100 cyclic tensile tests on a universal testing machine (Model 5848, Instron, Norwood, MA, USA) to obtain stress–strain curves during cyclic stretching. Young’s modulus (E), defined as the ratio of stress (σ) to strain (ε), was derived from the slope of the stress–strain curve to further evaluate the mechanical strength of the hydrogel.

The catheter coating’s frictional characteristics were assessed with an friction and wear testing machine, using porcine bladder mucosa as the contact interface. Two samples from both the test and control groups were secured on a V-groove plate with a clamping device after 30 s of water immersion. PBS (pH = 7.3) was then injected into the reservoir as a lubricant until the samples were completely submerged. A 200 g standard slider was carefully placed on the samples and moved along the direction marked by an arrow at 100 mm/min *via* a sensor-connected rod, allowing measurement of the dynamic friction force and friction coefficient. The average friction coefficient was calculated from six replicate samples.

#### 2.5.5 Release profiles of MgO at different pH values

MgO@AAm/SA/TA coatings (approximately 20 mg) were immersed in 1 mL of PBS solutions (pH 7.4, 8.0, 8.5, and 9.0) and incubated at 37 °C with shaking at 50 rpm. At predetermined intervals (0.5, 1, 2, 4, 6, 12, 24, 48, and 72 h), 500 μL of supernatant was collected and replaced with an equal volume of fresh PBS. The concentration of characteristic MgO elements in the supernatant was quantified using inductively coupled plasma mass spectrometry (ICP-MS). The cumulative release was then calculated to establish the release profiles.

#### 2.5.6 Sustained release of AMPs

The release of LL-37 from the PL@SAMT/Mg coating was quantified with a Micro BCA protein assay kit. Freeze-dried, LL-37-loaded coating samples (5 mg) were immersed in 1 mL of PBS solutions at pH values of 7.4, 8, 8.5, and 9. These samples were subsequently centrifuged at 10,000 rpm for 10 min. All samples were then incubated at 37 °C in a constant temperature incubator. At designated intervals, 200 µL of supernatant was extracted and replaced with an equal volume of fresh PBS to maintain a constant total volume. The absorbance of the released LL-37 was measured at 562 nm using the BCA kit. The peptide release at each time point was quantified by establishing a standard curve.

### 2.6 Antibacterial properties *ex vivo*

*Staphylococcus aureus* (*S. aureus*) and *Escherichia coli* (*E. coli*) were selected as representative bacterial strains to evaluate the hydrogels' antibacterial efficacy. The coated samples were co-incubated with 1 mL of bacterial suspension (10^6^ CFU/mL) for 24 h. Subsequently, the bacterial suspensions were serially diluted, plated onto agar plates, and the number of colonies was counted to determine the bacterial survival rates. The bacterial survival rates was calculated according to the following formula:

$$\text{Bacterial survival ratio }\left(\%\right)=\frac{\mathrm{Cell}\mathrm{count}\;\mathrm{of}\;\mathrm{hydrogels}}{\mathrm{Cell}\;\mathrm{count}\;\mathrm{of}\mathrm{control}\mathrm{survivor}}\times 100\%$$

The morphology of the bacteria was examined by SEM at magnifications of ×10,000 and ×300,00, and images of the surviving bacteria were captured.

### 2.7 *Ex vivo* biocompatibility

#### 2.7.1 CCK-8 assay for cell viability determination

L929 cells and SV-HUC-1 urothelial cells were seeded in 96-well plates at a density of 5 × 10^3^ cells per well. Each cell type was divided into three groups. Coating extracts were prepared by immersing the corresponding coatings (20 µL coating volume per well equivalent) in DMEM medium supplemented with 10% FBS, which served as the extraction medium, followed by incubation at 37 °C for 24 h. The cells in each group were then treated with 20 µL of either the PL@SAMT coating extract, the PL@SAMT/Mg coating extract, or an equal volume of blank culture medium. After co-incubation for predetermined time intervals (1, 3, and 5 days), 10 µL of CCK-8 reagent was added to each well. Following a 2-h incubation period, the absorbance at 450 nm was measured using a microplate reader. Cell viability was calculated according to the following formula:

$$\text{Cell viability }\left(\%\right)=\left[\left(\text{As}-\text{Ab}\right)/\left(\text{Ac}-\text{Ab}\right)\right]\times 100$$

Here, As represents the absorbance of the sample (including the cell and coating extract solution of the CCK-8 solution); Ac is the absorbance of the control group (CCK-8 solution containing cells but without the coating extract solution); Ab is the absorbance of the blank group (CCK-8 solution without cells or coating extract solution).

#### 2.7.2 Live/Dead staining

L929 and SV-HUC-1 cells were seeded in 24-well plates (2 × 10^4^ cells/well), with each cell type divided into two groups. Each group was treated with 20 μL of either PDA/LL-37 coating extract or PL@SAMT/Mg coating extract. After co-incubation for predetermined time points (1, 3, and 5 days), the cells were stained using a Calcein-AM/PI staining kit. The ratio of live cells (green fluorescence) to dead cells (red fluorescence) was observed under a fluorescence microscope.

### 2.8 Detection of inflammatory cytokine secretion

RAW264.7 cells were seeded in 24-well plates at a density of 1 × 10^5^ cells per well and divided into three groups. Each group was treated with 20 μL of PL@SAMT coating extract, 20 μL of PL@SAMT/Mg coating extract, or 1 μg/mL lipopolysaccharide (LPS) to induce inflammation, respectively. After 24 h of incubation, the supernatants were collected. The concentrations of tumor necrosis factor-alpha (TNF-α) and interleukin-6 (IL-6) in the conditioned medium were measured using corresponding enzyme linked immunosorbent assay (ELISA) kits.

### 2.9 Cell adhesion assay

PDA/LL-37-coated and PL@SAMT/Mg-coated samples were placed in a 24-well plate, with uncoated catheters serving as controls. Each well was seeded with 1 × 10^5^ cells and incubated for 4 h. After incubation, non-adherent cells were removed by washing with PBS. The adherent cells were fixed with formaldehyde, stained with crystal violet, and counted under a microscope.

### 2.10 Animal experiments

A total of 20 female Sprague-Dawley (SD) rats (aged 6–8 weeks) were used in this study. The animals were acclimatized for approximately 3 days under controlled environmental conditions at 18 °C–22 °C.

To minimize the trauma area, medical catheter sheaths (24G, thermoplastic polyurethane) were selected as implants. Prior to the animal experiments, catheter sheaths for the coated and uncoated groups were prepared under aseptic conditions. The sample processing procedure was as follows: A 5 mm segment was excised from the tip of the indwelling needle sheath. The segments were then ultrasonically cleaned sequentially in ethanol and deionized water for 15 min each. For the uncoated samples, the excised segment was reconnected to the remaining catheter body and reattached to the needle assembly. For the coated samples, the 5 mm segment was immersed in the PL@SAMT/Mg monomer solution to form the coating, after which it was reassembled to its original configuration.

#### 2.10.1 Host response to sterile subcutaneously implanted PL@SAMT/mg-coated catheters

To ensure experimental feasibility and the scientific validity of the results, SD rats were divided into two groups (*n* = 5 per group) and implanted with either blank control catheters or experimental PL@SAMT/Mg-coated catheters. Anesthesia was induced in the rats *via* intraperitoneal injection of 0.10–0.20 mL of 5% isoflurane and maintained with 3% isoflurane. Following an abdominal skin incision, two 5-mm medical catheter samples were implanted subcutaneously in each rat. According to the sample dimensions, 5-mm incisions were made on both sides of the rat’s abdomen using surgical scissors. To facilitate comparison, both coated and uncoated catheters were implanted in the same rat. After disinfection with povidone-iodine, the skin was sutured, and the rats were housed under standard conditions. Five days post-implantation, the catheter implantation sites were photographed. The muscle tissue surrounding the implantation sites was then harvested and immersed in 4% paraformaldehyde fixative for 24 h. Subsequently, the tissues were rinsed under running water and dehydrated through a graded series of ethanol solutions (30%, 50%, 70%, 80%, 95%, and 100%). Following complete dehydration and xylene clearing, the tissue sections were embedded in paraffin. The muscle tissues from the implantation sites were stained using a Hematoxylin and Eosin (H&E) staining kit. The stained sections were observed and analyzed using a fluorescence microscope.

#### 2.10.2 *In Vivo* antibacterial activity

In the implant-associated bacterial infection model, catheters were prepared by immersion in a *S. aureus* suspension (1 × 10^6^ CFU/mL), incubation at 37 °C for 3 h, and repetition of this treatment. On day 5, the rats were euthanized using carbon dioxide at a displacement rate of 50% chamber volume per minute for 10.5 min to ensure irreversible euthanasia. After euthanasia, specimens from the implantation sites were collected and fixed in 4% paraformaldehyde (PFA) for 24 h at 4 °C. The inflammatory status at the implantation site was assessed macroscopically, and specimens were processed for histological analysis as described previously. The implanted catheter was then removed, immersed in 0.3 mL of sterile PBS for 5 min, and treated with ultrasonication. Following ultrasonic disruption, the samples underwent serial dilution and were plated on agar for colony counting. Bacterial counts in the samples were determined according to the JIS Z 2801 standard.

### 2.11 Statistical analysis

All numerical data are expressed as mean ± standard deviation. Differences between two groups were analyzed using the independent samples t-test, while differences among multiple groups were assessed by one-way analysis of variance (ANOVA) followed by Tukey’s *post hoc* test for multiple comparisons. All statistical analyses were performed using SPSS version 22.0 (IBM SPSS Inc., Chicago, IL, USA). A *p < 0.05 and **p < 0.01 were considered statistically significant, whereas *p* > 0.05 indicated no statistically significant difference.

## 3 Result and discussion

### 3.1 Fabrication of PL@SAMT/Mg coating

**FIGURE 1:**
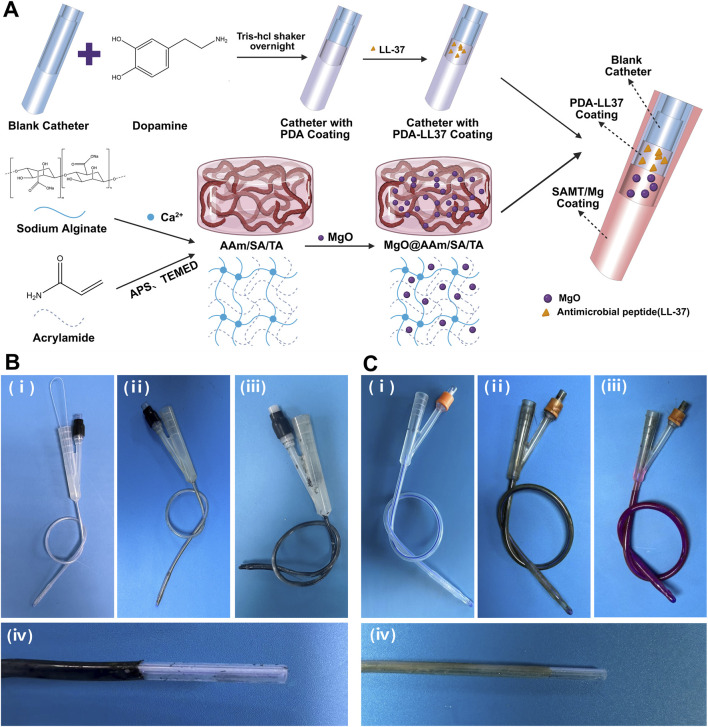
Structure of the urinary catheter coating. **(A)** Schematic diagram of the fabrication process of the PL@SAMT/Mg bilayer-coated catheter. **(B)** Physical images of the animal urinary catheter coating: (i) blank animal urinary catheter, (ii) animal urinary catheter coated with PDA/LL-37, (iii) animal urinary catheter coated with PL@SAMT/Mg, (iv) image showing the coating and internal structure of the animal urinary catheter. **(C)** Physical images of the human urinary catheter coating: (i) blank human urinary catheter, (ii) human urinary catheter coated with PDA/LL-37, (iii) stained human urinary catheter coated with PL@SAMT/Mg, (iv) image showing the coating and internal structure of the human urinary catheter.

We successfully fabricated the PL@SAMT/Mg bilayer-coated catheter (Figure 1A) and applied it to the surfaces of animal urinary catheters (Figure 1B) and human urinary catheters (Figure 1C), respectively.

### 3.2 Characterization of PL@SAMT/Mg coatings

**FIGURE 2:**
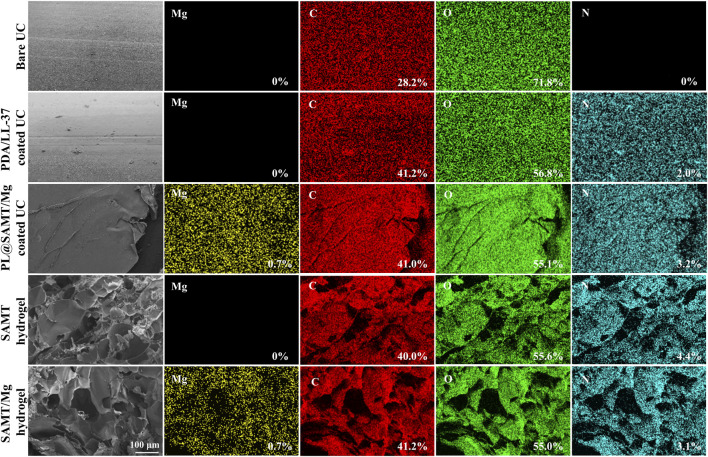
SEM and EDS elemental mapping images of blank urinary catheters, PDA/LL-37-coated catheters, PL@SAMT/Mg-coated catheters, SAMT hydrogels, and SAMT/Mg hydrogels (Magnification: ×150).

The fabricated PL@SAMT/Mg coatings were characterized in detail. SEM images revealed the microstructure of the coatings and hydrogels (Figure 2). Unlike the relatively smooth surface of the blank urinary catheter, the PDA/LL-37-coated catheter exhibited a rougher morphology with distributed PDA particles. The PL@SAMT/Mg-coated catheter displayed a bilayer hybrid structure, where granular PDA was located at the hydrogel base and MgO particles were uniformly dispersed across the hydrogel surface. These observations indicate that both the PDA/LL-37 and SAMT/Mg hydrogel bilayer coatings adhered tightly to the catheter. Higher-magnification SEM images further showed that the SAMT hydrogel possessed a three-dimensional porous network. EDS analysis detected a substantial increase in nitrogen content on the PDA/LL-37-coated catheter relative to the blank catheter (Figure 2). Magnesium was uniformly distributed across the surface of the PL@SAMT/Mg-coated catheter, corroborating the SEM results and further confirming the firm attachment of both coatings. Moreover, the Mg content in the SAMT/Mg hydrogel was significantly higher than in the SAMT hydrogel, verifying the successful loading of MgO particles.

In addition to qualitative morphological analysis, the PL@SAMT/Mg coating was further characterized by quantitative structural parameters. As shown in the macroscopic cross-sectional images in Supplementary Figure S1, the thickness of the hydrogel coating was measured to be approximately 150–200 μm, indicating the formation of a uniform and continuous coating layer on the catheter surface that is sufficient to provide lubrication and antibacterial functionality without excessive bulk. The porosity of the SAMT/Mg hydrogel was determined to be 81.97% based on SEM analysis (Supplementary Figure S2), confirming a highly porous three-dimensional network structure that facilitates water retention, hydration lubrication, and diffusion-controlled release of antibacterial agents. In addition, the MgO nanoparticles incorporated into the hydrogel exhibited an average particle size of approximately 50 nm, consistent with the specifications of the commercial MgO nanoparticles used in this study (XFNANO Materials Technology), which favors homogeneous dispersion within the hydrogel matrix and effective antibacterial activity. These quantitative results provide a more comprehensive and rigorous characterization of the PL@SAMT/Mg coating and support its stable lubricating performance and controllable antibacterial behavior.

**FIGURE 3:**
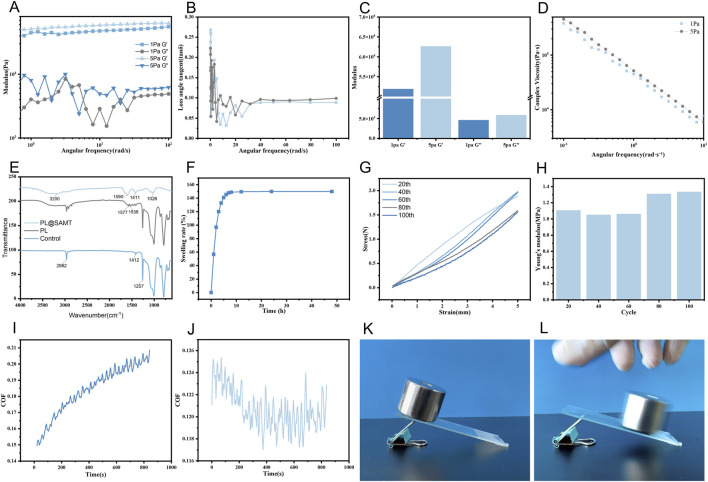
**(A)** Rheological frequency sweep of (AAm/SA/TA) hydrogels under oscillatory stresses of 1 and 5 Pa. **(B)** Loss tangent (tan δ) of the (AAm/SA/TA) hydrogel as a function of angular frequency. **(C)** Storage modulus (G′) and loss modulus (G″) of the (AAm/SA/TA) hydrogel under oscillatory stresses of 1 and 5 Pa. **(D)** Viscosity of the (AAm/SA/TA) hydrogel as a function of angular frequency. **(E)** FTIR spectrum of the blank catheter substrate, the PL layer, and the PL@SAMT hydrogel coating. **(F)** Swelling ratio of the (AAm/SA/TA) hydrogel as a function of time. **(G)** Stress–strain curve of the (AAm/SA/TA) hydrogel. **(H)** Young’s modulus of the (AAm/SA/TA) hydrogel during multiple cyclic tensile tests. **(I)** Friction coefficient of the (AAm/SA/TA) hydrogel over time before hydration. **(J)** Friction coefficient of the (AAm/SA/TA) hydrogel over time after hydration. **(K)** Inclined plane test of the (AAm/SA/TA) hydrogel before hydration. **(L)** Inclined plane test of the (AAm/SA/TA) hydrogel after hydration.

The rheological properties of the (AAm/SA/TA) hydrogel were characterized through frequency sweep measurements. As shown in Figure 3A, across the frequency range of 0.1–100 rad/s, the storage modulus (G′) of the hydrogel consistently exceeded the loss modulus (G″) by approximately one order of magnitude, indicating a stable and rigid coating structure (Geng et al., 2022). Both the G′ and G″ curves remained relatively flat with increasing frequency, reflecting a stable hydrogel network with a long relaxation time that effectively resists deformation over various time scales. Under applied stresses of 1 Pa and 5 Pa, G′ maintained a high value between 10^4^ and 10^5^ Pa, demonstrating that the multiple cross-linked network—formed by covalent acrylamide chains, ionic alginate associations, and hydrogen bonds from tannic acid—yields a robust three-dimensional architecture (Zhang Y. et al., 2023; Zhang X. et al., 2023). This mechanical integrity provides a solid foundation for the hydrogel’s function as a durable coating.

The viscoelastic equilibrium of the (AAm/SA/TA) hydrogels was further characterized using angular frequency–loss tangent plots. The loss tangent (tan δ) represents the ratio of the viscous component (G″) to the elastic component (G′). Materials with tan δ < 1 display primarily elastic behavior, whereas those with tan δ > 1 are dominated by viscous dissipation (Murru et al., 2022). As shown in Figure 3B, the loss tangent values remain below 0.25 across the entire frequency range and fall under 0.1 in most high-frequency regions, indicating an elastic-dominated hydrogel state more characteristic of a solid than a liquid (Xu et al., 2023). This strong elastic character ensures that the coating maintains its shape *in vivo* and provides stable mechanical support, rather than undergoing viscous flow. The slight increase in tan δ observed at low frequencies (∼0.1 rad/s) likely reflects the slow motion or rearrangement of extended chain segments or weaker physical crosslinks, such as ionic or hydrogen bonds, though this does not undermine the hydrogel’s overall stability.

Figure 3C provides critical evidence supporting the validity of the rheological tests and the reliability of the hydrogel in practical applications. As the oscillatory stress increases from 1 to 5 Pa, only a minor change in G′ occurs. This result confirms that both frequency sweep tests were performed within the material’s linear viscoelastic region (LVER) (El Bitouri, 2023). The hydrogel’s microstructure thus remained intact during testing, and the data accurately reflect its intrinsic properties (El Bitouri, 2023). More importantly, a broad LVER indicates that the hydrogel coating can endure stress fluctuations—such as those from urine flow or tissue friction—in real-world use without yielding structurally or losing mechanical performance, ensuring long-term reliability in clinical settings (Ma et al., 2020).

Shear tests further characterized the hydrogel’s flow behavior to assess its processability and practical utility. As shown in Figure 3D, the hydrogel’s complex viscosity (η*) dropped markedly with rising angular frequency, reflecting typical shear-thinning behavior (Correa et al., 2021). This property offers dual advantages: on one hand, during coating processes such as dip-coating or spraying, the elevated shear rates reduce viscosity, thereby facilitating the handling and uniform application of the pre-gel solution (Lao et al., 2024). On the other hand, under *in vivo* conditions, when the urinary catheter undergoes sudden deformation or high shear stress—such as during catheter insertion or changes in body position—the temporary reduction in viscosity helps dissipate mechanical stress, protecting the coating–tissue interface. Once shear is removed, viscosity quickly recovers, preserving the protective function of the coating (Wang et al., 2017).

The chemical structure of the blank catheter substrate, the PL layer, and the PL@SAMT hydrogel was systematically investigated by FTIR spectroscopy (Figure 3E). By comparing the FTIR spectral features of different samples, it can be concluded that PDA was stably grafted onto the catheter surface and that the antibacterial hydrogel was successfully loaded onto the PDA-modified catheter. The modified layers formed stable interfacial bonding with the substrate and between adjacent layers, without disrupting the intrinsic chemical structure of the catheter material. The FTIR spectrum of the control catheter exhibited only characteristic peaks of the polymeric substrate, including the saturated C–H stretching vibration at 2,962.25 cm^−1^, the C–H bending vibration at 1,412.12 cm^−1^, the C–O–C stretching vibration at 1,257.76 cm^−1^, and the C–C/C–O skeletal vibrations in the range of 700–1,007 cm^−1^. For the PL group, all substrate-related peaks were well preserved, while two additional peaks at 1,538.77 and 1,577.03 cm^−1^ appeared, corresponding to the aromatic C=C stretching vibrations of polydopamine, which are recognized as its characteristic signatures. The negligible shifts and stable intensities of the original substrate peaks indicate that PDA was immobilized *via* surface interactions without damaging the catheter matrix. Upon formation of the PL@SAMT hydrogel layer, several distinct characteristic absorption peaks were observed in the FTIR spectrum. Firstly, a broad peak observed around 3,200 cm^−1^ is attributed to the overlapping O–H stretching vibrations of sodium alginate and tannic acid, as well as the N–H stretching vibration of polyacrylamide. The breadth of this peak is indicative of strong hydrogen bonding within the hydrogel. Meanwhile, the peak at 1,590 cm^−1^ corresponds to the amide II band of polyacrylamide, confirming the successful involvement of both polyacrylamide and sodium alginate in the cross-linking process. Secondly, the peak at 1,411.67 cm^−1^ is assigned to the symmetric COO^−^ stretching vibration of sodium alginate or the C–N stretching vibration of polyacrylamide, consistent with the ionic cross-linking of sodium alginate. Furthermore, the peak at 1,026 cm^−1^ represents the C–O stretching vibration, primarily originating from the ether linkage (C–O–C) and the alcoholic hydroxyl group (C–OH) in sodium alginate and tannic acid. This peak verifies the presence of the polysaccharide backbone in the PL@SAMT hydrogel.

Although hydrogels can effectively absorb urethral secretions to prevent bacterial infection, excessive water absorption can lead to hydrogel swelling, making catheterization more difficult (Liang et al., 2019; Yong et al., 2019). Therefore, a low water absorption rate of the catheter coating is crucial. The anti-swelling properties of the samples were evaluated by measuring the swelling ratio of (AAm/SA/TA) hydrogels. As shown in Figure 3F, when the immersion time reached 10 h, the hydrogels reached an equilibrium swelling state, with the swelling ratio maintained at approximately 150%. This indicates that the hydrogel coating possesses a relatively dense network structure, where interconnected pores allow the hydrogel to retain some water molecules without causing excessive swelling. The anti-swelling characteristics of the (AAm/SA/TA) hydrogel ensure shape stability of the hydrogel-coated catheter in the urethral environment, thereby reducing issues such as catheter blockage and coating detachment caused by hydrogel expansion.

To further evaluate the stability of the (AAm/SA/TA) hydrogel, its mechanical properties under cyclic loading were examined. As shown in Figure 3G, the stress–strain curves of the (AAm/SA/TA) hydrogel at the 20th, 40th, 60th, 80th, and 100th cycles exhibited highly similar profiles, all characterized by nearly linear elastic deformation. A similar trend was observed in the variation of Young’s modulus with the number of cycles. Figure 3H indicates that Young’s modulus did not show significant regular changes during multiple cycles, with only minor fluctuations observed at higher cycle numbers. This may be attributed to the highly uniform internal molecular structure of the (AAm/SA/TA) hydrogel in terms of composition, grain size, and phase distribution. As a result, cyclic stress was insufficient to induce notable microstructural evolution, and the binding energy of the cross-links remained largely unchanged under cyclic loading, thereby maintaining the stability of Young’s modulus and stress response (Kuběna et al., 2015).

The fluid lubrication process is primarily influenced by viscosity. Within a certain range, a higher viscosity corresponds to a better lubricating effect of the hydrogel (Hu et al., 2020). Figure 3I shows the friction coefficient–time curves of the PL@SAMT/Mg coating before and after hydration. When the PL@SAMT/Mg coating was dry, the surface friction coefficient of the catheter gradually increased over time. However, after the addition of water to the coating (Figure 3J), the surface friction coefficient of the catheter gradually decreased over time and eventually stabilized, indicating that the surface of the PL@SAMT/Mg-coated catheter transitioned from an adhesive to a lubricating state upon hydration. This phenomenon occurs because hydrogel networks are highly effective at absorbing water and reducing friction (Liu et al., 2022; Lin et al., 2020). The results of the inclined plane test (Figures 3K,L) demonstrated that, under dry conditions, an object adhered to the surface of a PL@SAMT/Mg-coated glass slide and did not slide, whereas under wet conditions, the object slid naturally down the slope. Therefore, the PL@SAMT/Mg-coated catheter can provide effective lubrication in the moist environment of the urethra.

### 3.3 *In vitro* cytocompatibility of PL@SAMT/Mg coating

**FIGURE 4:**
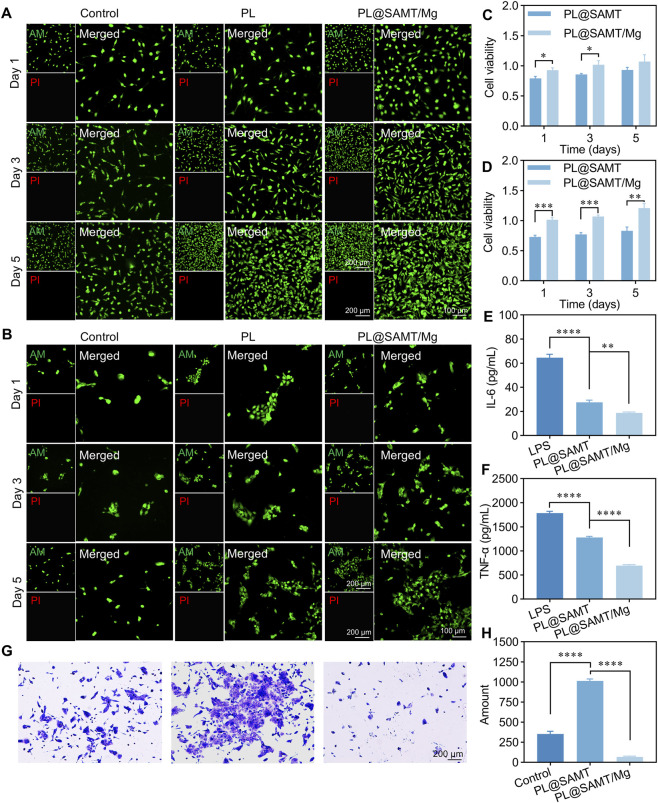
Cytocompatibility evaluation of the PL@SAMT/Mg coating. **(A)** Live/Dead staining of **(A)** L929 cells and **(B)** SV-HUC-1 cells after incubation with extracts of the PL@SAMT/Mg coating for 1, 3, and 5 days. **(C)** Cell viability of **(C)** L929 cells and **(D)** SV-HUC-1 cells after incubation with extracts of the PL@SAMT and PL@SAMT/Mg coatings for 1, 3, and 5 days, as assessed by the CCK-8 assay (*n* = 3) (*p < 0.05; **p < 0.01; ***p < 0.001). **(E)** Levels of **(E)** IL-6 and **(F)** TNF-α in RAW264.7 cells after 24-h incubation with extracts of the PL@SAMT/Mg coating, as determined by ELISA (*n* = 3) (**p < 0.01; ****p < 0.0001) **(G)** Crystal violet staining of SV-HUC-1 cells seeded on blank catheters, PDA/LL-37-coated catheters, and PL@SAMT/Mg-coated catheters after 4 h of culture, followed by washing and fixation. **(H)** Number of adherent SV-HUC-1 cells on blank catheters, PDA/LL-37-coated catheters, and PL@SAMT/Mg-coated catheters (*n* = 3). (****p < 0.0001).

Excellent cell compatibility is a crucial factor in the design of novel urinary catheter coatings. First, live/dead staining was employed to evaluate the biocompatibility of the catheter coatings. After 5 days of culture, L929 and SV-HUC-1 cells were stained with fluorescent probes for live and dead cells. Live cells exhibited green fluorescence due to calcein-AM staining, whereas dead cells displayed red fluorescence due to propidium iodide (PI) staining. As shown in Figures 4A,B, the green fluorescence intensity of both L929 and SV-HUC-1 cells in the PL@SAMT/Mg coating group was higher than that in the control and PL groups. Furthermore, almost no dead cells were observed in the PL@SAMT/Mg coating group, indicating that the prepared catheter coating is non-cytotoxic. Subsequently, a more comprehensive assessment of the cytotoxicity of the PL@SAMT/Mg coating was conducted using the CCK-8 assay. Figures 4C,D shows that the OD_450_ values for both L929 and SV-HUC-1 cells in the PL@SAMT/Mg coating group were significantly higher than those in the PL@SAMT coating group. This result indicates that the cell viability in the PL@SAMT/Mg coating group was significantly greater than that in the PL@SAMT coating group, further confirming the excellent biocompatibility of the PL@SAMT coating.

Catheter-associated infections can induce local inflammation of the urinary tract, and the tissue damage caused by inflammation may further exacerbate infection and pain (Nickel et al., 2024; Liu and Kamperman, 2025). Among inflammatory mediators, cytokines such as TNF-α and IL-6 act as key regulators of inflammation and promote inflammatory responses by modulating the inflammatory cascade (Deng et al., 2022; Saini et al., 2017). Blocking the release of pro-inflammatory factors TNF-α and IL-6 helps suppress persistent inflammatory states, thereby alleviating catheter-associated infections and pain. Therefore, the effect of the catheter coating on the expression of TNF-α and IL-6 was analyzed. As shown in Figures 4E,F, compared with the LPS-treated group and the PL@SAMT coating group, the TNF-α and IL-6 levels in the PL@SAMT/Mg coating group were significantly decreased, confirming the potent anti-inflammatory capacity of the PL@SAMT/Mg coating and indicating that the release of MgO significantly enhanced the anti-inflammatory performance of the coating. Cheng et al. (2025) reported similar findings, demonstrating that MgO@ABs (MgO@Apoptotic Bodies) effectively suppressed the inflammatory response by reducing the secretion level of TNF-α.

The surface of indwelling urinary catheters is prone to adhesion of urethral or bladder epithelial cells, which not only facilitates bacterial adhesion and biofilm formation but may also cause pain and discomfort (Barford et al., 2008). Therefore, the anti-adhesion properties of catheter coatings are crucial for their applications in infection prevention and lubrication. To further elucidate the anti-adhesion performance of the PL@SAMT/Mg coating, samples with different coatings were placed in 24-well plates, and SV-HUC-1 cells were seeded onto their surfaces and cultured for 4 h. As shown in Figures 4G,H, compared with the control and PL-coated groups, the number of adherent cells was significantly reduced in the PL@SAMT/Mg-coated group, indicating excellent anti-adhesion properties of the PL@SAMT/Mg coating. In summary, the PL@SAMT/Mg coating exhibits favorable cytocompatibility and anti-inflammatory functions, along with outstanding anti-adhesion performance.

### 3.4 The antibacterial activity of PL@SAMT/Mg coating

**FIGURE 5:**
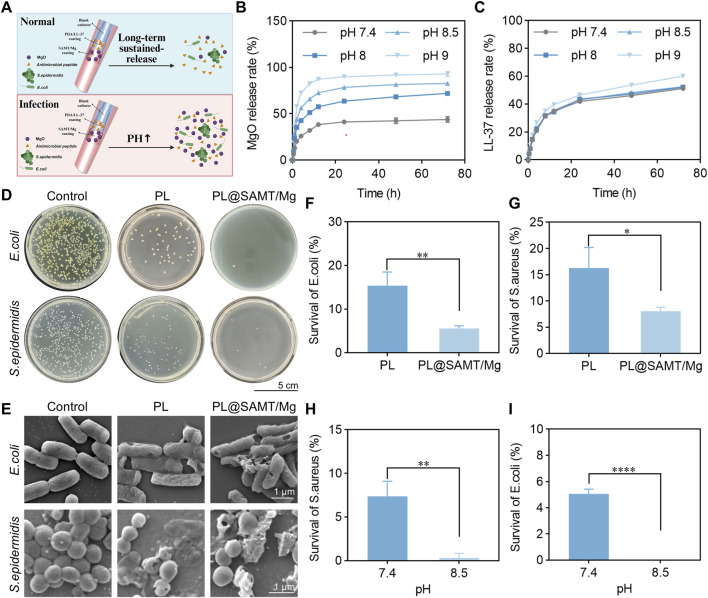
Antibacterial performance of the PL@SAMT/Mg coating. **(A)** Schematic diagram illustrating the release profiles of AMPs and MgO from the PL@SAMT/Mg coating. **(B)** Release curves of **(B)** MgO and **(C)** LL-37 over time at different pH values (*n* = 3). **(D)** Photographs of *E. coli* (top) and *S. aureus* (bottom) after 24 h of co-culture with the blank control, PDA-LL37 coating, and PL@SAMT/Mg coating, respectively (scale bar: 5 cm). **(E)** SEM images of *E. coli* (top) and *S. aureus* (bottom) after 24 h of co-culture with the blank control, PDA-LL37 coating, and PL@SAMT/Mg coating, respectively (scale bar: 1 µm). **(F)** Survival rates of **(F)** *E. coli* and **(G)** *S. aureus* after 24 h of co-culture with the PDA-LL37 coating and PL@SAMT/Mg coating, respectively (*n* = 3). (*p < 0.05; **p < 0.01). **(H)** Survival rates of **(H)** *S. aureus* and **(I)** *E. coli* at pH 7.4 and pH 8.5, respectively (*n* = 3). (**p < 0.01; ****p < 0.0001).

Long-term indwelling of urinary catheters readily facilitates bacterial adhesion and subsequently induces urinary tract infections. Therefore, excellent antibacterial activity of the catheter coating is crucial for suppressing urinary tract infections (Liu and Kamperman, 2025). The multi-antibacterial functionality of the PL@SAMT/Mg composite coating primarily relies on the synergistic effect of antimicrobial peptides (AMPs) and magnesium oxide (MgO). On one hand, the distinct release profiles of AMPs and MgO can adapt to the complex and dynamic microenvironment of urinary tract infections. As shown in Figures 5A–C, the release rate of LL-37 is relatively slow, with no significant difference in release percentage under varying pH conditions, indicating that LL-37 is sufficiently released under the normal physiological urethral condition at pH 7.4 to exert routine bactericidal effects. In contrast, MgO exhibits a faster release rate, with a significantly increased release percentage in alkaline environments, demonstrating a responsive release characteristic to the elevated pH caused by urinary tract infection. This pH-responsive swelling behavior, which facilitates accelerated MgO release, is fundamentally governed by the ionization of specific functional groups within the SAMT hydrogel network. Under alkaline conditions, the carboxyl groups (-COOH) of SA and AAm undergo deprotonation to form negatively charged carboxylate anions (−COO^−^) (Sabbagh and Muhamad, 2017; Wang et al., 2024). Similarly, the abundant phenolic hydroxyl groups (−OH) on TA are also susceptible to deprotonation, acquiring negative charges (Liu et al., 2025). The collective generation of these negative charges introduces strong electrostatic repulsion between the polymer chains, causing the hydrogel network to expand (Sabbagh and Muhamad, 2017). Concurrently, the increased osmotic pressure within the gel, driven by the need to balance these fixed charges with counter-ions from the surrounding medium, leads to substantial water influx and pronounced hydrogel swelling (Sabbagh and Muhamad, 2017). This expanded network structure creates larger diffusion pathways and reduces barriers, thereby accelerating the release of embedded MgO nanoparticles. Thus, the combination of the preventive long-term sustained-release mode of AMPs during the uninfected stage and the rapid responsive release mode of MgO upon infection not only achieves comprehensive bactericidal efficacy but also reduces the risk of drug resistance and biotoxicity. On the other hand, the different bactericidal mechanisms of AMPs and MgO can further enhance the antibacterial effect. AMPs primarily rely on their positive charges to interact electrostatically with the negative charges on bacterial cell membranes, increasing membrane permeability and thereby exerting antibacterial activity. This unique mechanism makes it difficult for bacteria to develop resistance (Xuan et al., 2023). In contrast, MgO exerts broad-spectrum antibacterial effects by generating reactive oxygen species (ROS) such as superoxide radicals (•O_2_^−^) (Karthikeyan et al., 2021). Therefore, the distinct antibacterial mechanisms of AMPs and MgO complement each other, resulting in highly efficient combined antibacterial efficacy.

To evaluate the antibacterial capability of the urinary catheter coating, analyses were performed using Gram-negative *E. coli* and Gram-positive *S. aureus* through scanning electron microscopy (SEM) observation and bacterial survival rate determination. As shown in Figure 5D, compared with the control group, the PL@SAMT/Mg coating group exhibited a significant reduction in the number of bacterial colonies for both *E. coli* and *S. aureus*. Figure 5E reveals that *E. coli* in the control group grew well, displaying intact morphology and cell membranes, whereas the PL@SAMT/Mg coating group showed obvious rupture and damage to the cell membranes. SEM images of *S. aureus* indicated that the spherical structures remained intact in the control group, whereas obvious bacterial damage, manifested as varying degrees of bacterial fragmentation, was observed after the addition of the PL@SAMT/Mg antibacterial coating. Figures 5F,G demonstrate that the bacterial survival rate in the PL@SAMT/Mg coating group was significantly lower than that in the PL group, which is mainly attributed to the oxidative antibacterial activity generated by the release of MgO nanoparticles (MgONPs) (Karthikeyan et al., 2021). The bacterial survival rates at different pH values were further measured. As illustrated in Figures 5H,I, the bacterial survival rate at pH 8.5 was significantly lower than that at pH 7.4, which is closely associated with the increased swelling of the hydrogel in an alkaline environment, promoting the responsive release of MgO and enhancing antibacterial activity (Lei et al., 2025). In summary, the PL@SAMT/Mg coating represents an intelligent antibacterial material with strong antibacterial activity and pH-responsive antibacterial properties.

### 3.5 *In vivo* biocompatibility and antibacterial activity of urinary catheter coatings

**FIGURE 6:**
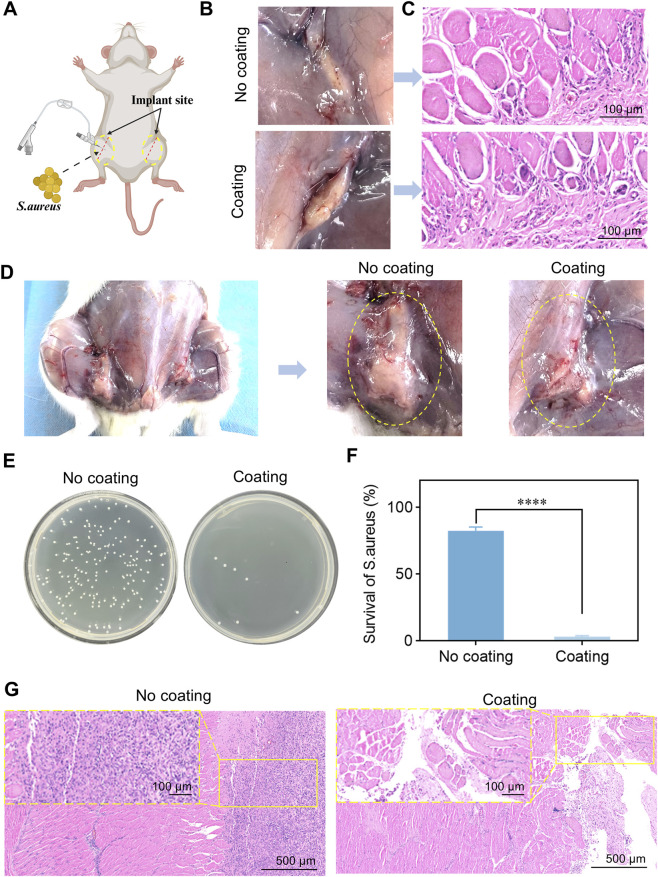
*In vivo* biocompatibility and antibacterial properties of the coating. **(A)** Schematic diagram of the rat subcutaneous implantation model. **(B)** Photographs of the catheter implantation sites. **(C)** Representative images of H&E staining (scale bar: 100 μm) at 5 days post-implantation. **(D)** Photographs showing the antibacterial performance at the catheter implantation sites after 5 days. **(E)** Distribution of *S. aureus* at the catheter implantation sites. **(F)** Survival rate of *S. aureus* at the implantation sites of uncoated and coated catheters (*n* = 5) (****p < 0.0001). **(G)** H&E staining of tissue sections surrounding the uncoated and coated catheters (scale bar: 500 μm).

The PL@SAMT/Mg coating demonstrated satisfactory cytocompatibility and antibacterial activity *ex vivo*. Therefore, as shown in Figure 6A, a rat subcutaneous implantation model was designed to further evaluate the *in vivo* biocompatibility and antibacterial activity of the coating. Figure 6B shows that no significant macroscopic inflammatory phenomena were observed at the implantation sites of either the bare or coated catheters. H&E staining was used to further examine the tissue inflammatory response at the implantation sites. As shown in Figure 6C, the tissue morphology surrounding the PL@SAMT/Mg-coated catheter implantation site appeared normal, with uniformly distributed nuclei at the periphery of individual cells, and no inflammatory cell infiltration was observed. Therefore, the PL@SAMT/Mg-coated catheter exhibits favorable biocompatibility.

To further investigate the *in vivo* antibacterial performance of the coating, catheters were immersed in a *S. aureus* suspension and re-implanted subcutaneously in rats. As shown in Figure 6D, a significant amount of pus was observed at the implantation site of the uncoated catheter, indicating severe bacterial infection. In contrast, no purulent infection was observed at the implantation site of the PL@SAMT/Mg-coated catheter. Bacterial plate counting results (Figure 6E) revealed extensive colonization of *S. aureus* on the surface of the uncoated catheter, whereas the number of bacterial colonies on the coated catheter surface was significantly reduced. Moreover, the antibacterial rate of the PL@SAMT/Mg-coated catheter was significantly higher than that of the uncoated catheter (Figure 6F), indicating that the PL@SAMT/Mg coating effectively kills bacteria colonizing the catheter surface. Bacterial infection is often accompanied by inflammatory cell infiltration (Abtin et al., 2014). H&E staining demonstrated diffuse and severe inflammatory cell infiltration in the tissue surrounding the uncoated catheter. In contrast, no inflammatory cell infiltration was observed around the PL@SAMT/Mg-coated catheter (Figure 6G). Additionally, we visually scored the degree of relative inflammation of the ducts according to a scoring system, where 0 means no inflammation and 4 means maximum inflammation (Freitas et al., 2022). In the uncoated catheter group, inflammatory cells were predominantly neutrophils, with diffuse infiltration covering approximately 40% of the area, accompanied by significant tissue edema, meeting the criteria for a “score of 3.” The final average score was 3.2 (*n* = 5). In the PL@SAMT/Mg coating group, only a small number of scattered lymphocytes were observed, with an infiltration area of <5% and normal tissue morphology, meeting the criteria for a “score below 1.” The final average score was 0.8 (*n* = 5). The scores between the two groups were compared using an independent samples *t*-test (*t* = 12.68, *p* < 0.0001), revealing a statistically highly significant difference, which further confirmed the anti-inflammatory efficacy of the coating. In summary, the PL@SAMT/Mg coating exhibits favorable biocompatibility and significantly reduces the risk of catheter-related infection.

### 3.6 Comparative performance analysis of PL@SAMT/Mg and contemporary antibacterial catheter coating

Compared with recently reported antibacterial catheter coatings, including silver-based systems, single pH-responsive coatings, and AMP-loaded coatings, the PL@SAMT/Mg coating demonstrates integrated advantages in antibacterial efficiency, biosafety, long-term stability, and lubricating performance. Conventional silver-based coatings rely on the release of silver nanoparticles (Ag NPs) for antibacterial activity. However, Ag NPs are susceptible to oxidation and aggregation, which significantly diminishes their antibacterial efficacy, and even when incorporated into hydrogel matrices, concerns remain regarding Ag+ accumulation-induced cytotoxicity, insufficient mechanical strength, and excessive coating swelling (Yang et al., 2024; Bai et al., 2023). In contrast, both AMPs and MgO used in the PL@SAMT/Mg system exhibit inherently low cytotoxicity, and their controlled release minimizes continuous exposure to metal ions while maintaining effective antibacterial performance. Compared with single pH-responsive systems, such as the PVA–Eudragit S100 bilayer coating that releases bacteriophages only when urine pH is elevated by *Proteus* infection and shows little antibacterial activity under normal physiological conditions or in the presence of urease-negative bacteria (Liu and Kamperman, 2025), the PL@SAMT/Mg coating provides sustained baseline antibacterial protection through the long-term, slow release of AMPs mediated by the polydopamine layer. When infection induces an increase in pH, the SAMT/Mg hydrogel layer further responds by accelerating MgO release, thereby enhancing antibacterial intensity. This combination of continuous protection and infection-triggered reinforcement broadens the range of effective antibacterial scenarios. Furthermore, although AMP-based coatings such as the AMP-EC-PCL system can achieve sustained AMP release for several days, they are typically hydrophobic, lack lubricating properties, and do not incorporate environmentally responsive antibacterial regulation (Low et al., 2021). By comparison, the hydrophilic SA/AAm/TA hydrogel network in the PL@SAMT/Mg coating endows the catheter surface with excellent and durable lubricity, while the pH-responsive release of MgO enables intelligent modulation of antibacterial activity, improving both targeted infection control and patient comfort. Overall, the bilayer design and synergistic release of AMPs and MgO provide the PL@SAMT/Mg coating with comprehensive improvements over existing antibacterial catheter coatings, representing an innovative strategy for next-generation catheters that combine intelligent antibacterial function, sustained lubrication, and favorable biocompatibility.

### 3.7 Clinical translation potential and degradation performance of PL@SAMT/Mg coatings

The clinical translation feasibility and degradation behavior of the PL@SAMT/Mg coating were analyzed to assess its prospects for practical application. The raw materials—SA, AAm, MgO, and LL-37—are commercially available at low cost, and the dip-coating and UV cross-linking process is compatible with existing catheter production lines, requiring no specialized equipment. Sterilization, a critical factor for clinical use, was achieved *via* γ-irradiation (25 kGy), which did not significantly compromise the coating’s antibacterial or lubricating properties. A preliminary cost estimate indicates an additional expense of only about $0.5 per catheter, a clinically acceptable increment. *In vitro* degradation testing in PBS (pH 7.4, 37 °C) showed a mass loss of approximately 50% over 8 weeks, while subcutaneous implantation in rats resulted in ∼35% degradation after 4 weeks with no evident accumulation. The degradation products, including renally excreted Mg^2+^, amino acids from the antimicrobial peptides, and polysaccharide fragments from SA/AAm/TA, are biocompatible and metabolically tractable. These results demonstrate the coating’s favorable biosafety and support its potential for clinical translation as a durable, intelligent antibacterial lubricant for urinary catheters.

## 4 Conclusion

This study successfully developed a medical catheter coating that integrates controllable antibacterial activity and sustained lubrication functions. The PL@SAMT/Mg coating exhibited excellent mechanical properties, lubricity, and antibacterial performance. *Ex vivo* experiments demonstrated that the coating on the urinary catheter was durable and possessed favorable lubricating characteristics, enabling stable adhesion to the catheter surface and achieving long-lasting lubrication. Antibacterial tests revealed that the coating exhibited pH-responsive antibacterial properties, showing significant antibacterial effects against *Staphylococcus aureus* and *Escherichia coli*. Furthermore, the coating also demonstrated good cytocompatibility and biocompatibility, not only exhibiting effective anti-adhesion performance but also inhibiting bacterial growth and the associated inflammatory response around the implantation site. Therefore, this novel composite coating provides an innovative and effective strategy for the surface modification of urinary catheters with integrated intelligent antibacterial and stable lubrication functions, demonstrating promising application prospects in reducing the risk of iatrogenic catheter-associated infections and improving patient comfort.

## Data Availability

The original contributions presented in the study are included in the article/Supplementary Material, further inquiries can be directed to the corresponding authors.
