# Supplementary figures and images for: A pH-Triggered antibacterial and lubricating dual-function hydrogel coating for infection-resistant urinary catheters
Source: Front Bioeng Biotechnol. 2026 Jan 21;14:1751442. doi: 10.3389/fbioe.2026.1751442 (PMC12868141; doi:10.3389/fbioe.2026.1751442)

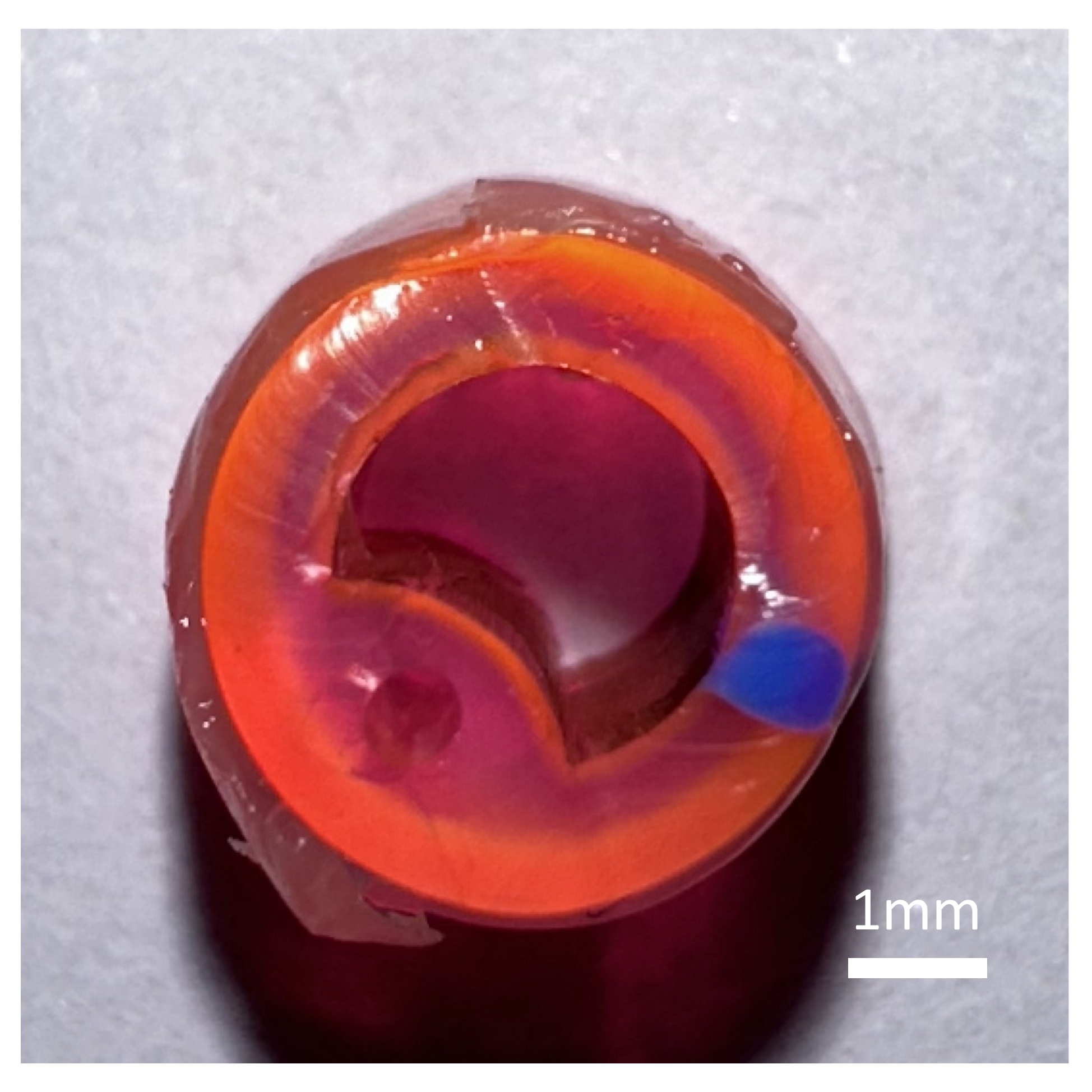

Supplement: Supplementary file 2 [file Image1.tif]
